# Molecular docking study of various *Enterovirus—A71* 3C protease proteins and their potential inhibitors

**DOI:** 10.3389/fmicb.2022.987801

**Published:** 2022-09-29

**Authors:** Tran Thao Vy Le, Phuc-Chau Do

**Affiliations:** ^1^School of Biotechnology, International University, Ho Chi Minh City, Vietnam; ^2^Vietnam National University, Ho Chi Minh City, Vietnam

**Keywords:** hand-foot-mouth disease, *Enterovirus–A71*, 3C protease, FIOMC, Hesperidin, Rupintrivir, broad-spectrum, antiviral agents

## Abstract

Hand, foot, and mouth disease (HFMD) is a common infection that primarily affects children in preschool and kindergarten; however, there is yet no vaccination or therapy available. Despite the fact that current research is only focused on numerous strains of *Enterovirus—A71 (EV-A71)* 3C protease (3C^pro^), these investigations are entirely separate and unrelated. Antiviral agents must therefore be tested on several EV strains or mutations. In total, 21 previously reported inhibitors were evaluated for inhibitory effects on eight *EV-A71* 3C^pro^, including wild-type and mutant proteins in this study, and another 29 powerful candidates with inhibitory effects on *EV-A71* were investigated using the molecular docking approach. This method is to determine the broad-spectrum of the antiviral agents on a range of strains or mutants because the virus frequently has mutations. Even though Rupintrivir is reported to pass phase I clinical trial, 4-iminooxazolidin-2-one moiety (FIOMC) was shown to have a broader anti-3C^pro^ spectrum than Rupintrivir. Meanwhile, Hesperidin possessed a better 3C^pro^ inhibitory capability than FIOMC. Thus, it could be considered the most promising candidate for inhibiting various strains of *EV-A71* 3C^pro^ proteins in the newly anti-EV compounds group. Furthermore, the mutation at E71A has the most significant impact on the docking results of all ligands evaluated. Future *in vitro* experiments on Hesperidin’s ability to inhibit 3C^pro^ activity should be conducted to compare with FIOMC’s *in vitro* results and validate the current *in silico* work.

## Introduction

Hand, foot, and mouth disease (HFMD) is a prevalent illness in preschool and kindergarten children. This global chronic health threat has recently produced substantial outbreaks in the Asia-Pacific area. The disease is caused by a group of viruses, mainly *Coxsackievirus* (*CV*) *A16* and *Enterovirus* (*EV*) *A71* (Aswathyraj et al., 2016). These viruses are mainly transmitted through the gastrointestinal tract (saliva, blisters, and feces of the sick person) and predominantly occur in the ages under five. According to the Ministry of Health, the disease is divided into four levels with different manifestations. The serve symptoms, such as neurological complications and death from level 2 or above, must have inpatient treatment and monitoring at the hospital. HFMD currently has no vaccine and specific treatment, only supportive treatment (with antibiotics if there is superinfection) for patients. Since the disease’s pathogenesis was not fully understood, the first *EV-A71* outbreak was discovered in Hubei Province in 1987, according to the Chinese epidemic statistics. Approximately 13.7 million cases of HFMD were reported between 2008 and 2015 in China, as determined by an extensive nationwide improved surveillance system for the disease developed more than 20 years later. These cases included 123, 261 severe cases, 3,322 fatalities, and the majority of reported cases in children under the age of five (Zheng et al., 1995; Qiu, 2008; Yang et al., 2009, 2017; Xing et al., 2014; Zhuang et al., 2015; Takahashi et al., 2016; Huang et al., 2018; Puenpa et al., 2019). Other Asian regions (e.g., Singapore, Japan, and Malaysia) also witnessed many similar breakouts, urgently seeking treatments against EV strains in Asia (Wu et al., 2010; Koh et al., 2016, 2018). However, it has been reported that *EV-A71* in Europe and America does not attract much attention due to their small number, infrequent occurrence, and low mortality rate, leading to less research conducted on vaccines or therapeutics on this EV species in these countries; meanwhile, the basic research on a different mechanism of action was focused (Esposito and Principi, 2018).

*EV-A71*, a member of the *Picornaviridae* family, is a positive single-stranded RNA virus with a genome size of around 7.5 kb that produces a large polyprotein separated into P1, P2, and P3 components. P1 typically encodes four structural capsid proteins (VP1–VP4) that aid cellular entry and viral genome transport into the host cell’s cytoplasm, whereas P2 and P3 productions yield seven non-structural proteins, supporting viral RNA replication, namely, 2A protease (2A^pro^), 2B, 2C, 3A, 3B, 3C protease (3C^pro^), and 3D polymerase (3D*^pol^*) (Bedard and Semler, 2004). There is a myriad of treatments worldwide that target different proteins of *EV-A71*. One of the most common research proteins is 3C^pro^, which inhibits its enzymatic function of cleaving viral polypeptides during viral infection. This 3C^pro^ has been found similarity with 3CL^pro^ in *Severe Acute Respiratory Syndrome - Coronavirus - 2* (SARS-CoV-2) in both structure and function. Thus, it gathers much attention in research to treat the diseases (Marjomäki et al., 2021).

In the pharmaceutical industry, bioactive substances are popular for treating various health issues such as inflammation, cancer, and infections (Behl et al., 2021). The development of antiviral treatments for SARS, influenza A, and EV has sparked attention to phytochemicals extracted from various natural sources (Attia et al., 2020). Since the outbreak of *EV-A71* in Taiwan in 1998, many bioactive compounds, including alkaloids, flavonoids, and terpenes, have been researched by *in vitro* studies to identify their potential antiviral activity (Lin et al., 2012; Wang et al., 2014a,b; Cao et al., 2016; Yao et al., 2018). Besides, several synthetic drugs have also been synthesized to inhibit *EV-A71* 3C^pro^ activity (Kuo et al., 2008; Wang et al., 2011, 2017, 2022; Tan et al., 2013; Ma et al., 2016; Zeng et al., 2016; Lin et al., 2019; Xu et al., 2021). However, viruses have a high mutation coefficient and produce many different strains in a short time, leading to drug resistance. Moreover, current research has solely been focused on several strains of *EV-A71* 3C^pro^, and these studies are individual and independent. Thus, testing antiviral drugs on a variety of *EV* strains is necessary. Besides, infectious disease research requires high facilities and skilled research expertise. Prototype testing of different drugs on young children to assess treatment effectiveness is another challenge for medical practitioners because it is extremely dangerous and impractical.

Thus, this paper aims to evaluate the inhibitory effects of 21 published ligands (flavonoids and Rupintrivir derivatives) by the molecular docking method. The experiment could then provide information on broad-spectrum antiviral agents against both wild-type and mutated proteins of *EV-A71* 3C^pro^. The results were evaluated with previous *in vitro* research based on its active binding pocket. In addition, this study investigated the best-scored ligands’ interactions deeply. We also used the results to analyze the *EV-A71* 3C^pro^ inhibitory property of another 29 potent inhibitors, which were reported as the candidates for HFMD treatment.

## Materials and methods

### Preparation of protein structures

The structure of wild-type and mutated strains of 3C^pro^ proteins was obtained from the Research Collaboratory for Structural Bioinformatics (RCSB) Protein Data Bank in PDB format and displayed in Table 1. Then, the unnecessary chains of these protein structures were removed before going through an energy minimization step for reconfiguring proteins favorably with the proper molecular arrangement in space using the University of California, San Francisco (UCSF) Chimera program version 1.16 (Pettersen et al., 2004). The parameter was set as AMBERff14SB force field, 1,000 steepest descent steps (SDS), 100 conjugate gradient steps (CGS), and 0.2 Å for the size for SDS and CGS. Docking investigations were performed on the structures that had been minimized.

**TABLE 1:** Data collection of 3C^pro^ proteins used in this study and their relevant information.

No.	PDB ID	Resolution	Strain	Isolated year	Mutation(s)	Box sizes (x; y; z)	Center grids (x; y; z)
1	5C1U (Zhang et al., 2016)	1.49 Å	E20041040-TW-CDC	2004	Wild type	102; 104; 110	4.579; −14.545; −8.009
2	3SJO (Lu et al., 2011)	1.70 Å	SZ/HK08-06	2008	Wild type	122; 106; 110	2.366; 21.993; 5.492
3	3SJK (Lu et al., 2011)	2.10 Å	AH08/06	2008	Wild type	118; 116; 104	14.623; 18.649; 0.099
4	5GSO (Wang et al., 2017)	2.60 Å	BJ/CHN/2008	2008	Wild type	126; 116; 120	−28.619; 3.732; 100.047
5	5GSW (Wang et al., 2017)	3.19 Å	BJ/CHN/2008	2008	N69S	116; 118; 110	10.528; −0.362; 20.879
6	3QZQ (Wang et al., 2011)	1.70 Å	BJ/CHN/2008	2008	E71D	116; 114; 112	50.475; 15.501; 1.953
7	3QZR (Wang et al., 2011)	1.04 Å	BJ/CHN/2008	2008	E71A	108; 108; 126	3.456; −1.839; 4.344
8	7DNC (Dai et al., 2022)	1.17 Å	Clone 122F	2008		126; 104; 100	−23.396; 25.108; -1.809

### Preparation of ligand structures

In total, 50 ligands were discovered as prospective inhibitors in the published research, and their structures were retrieved in the structure data file (SDF) from the PubChem database (Supplementary Material 1; Kim et al., 2019, 2021). These 50 ligands comprised 21 ligands proved as 3C^pro^ inhibitors by experimental methods and 29 ligands studied as drug candidates for *EV-A71*. The ligands in SDF format were converted to PDB format using Open Babel version 2.4.0 for the docking experiment (O’Boyle et al., 2011). Regarding ligands with no structure found, they were drawn using the Chemdraw 2014 and subjected to 3D conformations before converting to PDB format by the Open Babel tool. All the ligands underwent the energy minimization step for configuration optimization using the UCSF Chimera version 1.16 tool with the same parameters used for proteins. After that, docking investigations were performed on the minimized structures.

### Molecular docking

AutoDock tools or MGL tools package version 1.5.7 were used to prepare grid boxes for proteins and ligands before docking (Morris et al., 2009). All 3D structures of chosen proteins collected from the energy minimization step continuously went through the addition of polar hydrogen atoms, the computation of Kollman charge, and the assignment of AD4 type for the targets. The proteins were produced in Protein Data Bank, Partial Charge (Q), and Atom Type (T) format (PDBQT) format. Likewise, the preparation of ligands after the energy minimization step started with detecting and choosing the root atom before exporting them in the PDBQT format. The grid box was set with a default value of 0.375 Å spacing, providing the residual coordinates for the x, y, and z dimensions (Table 1). Autodock Vina version 1.1.2, which is a computational docking program, uses a scoring function to anticipate the docking scores between ligands and the biological targets (Trott and Olson, 2009; Pantsar and Poso, 2018; Eberhardt et al., 2021). The energy range was within 4 Kcal/mol, and the number of modes for the individual docking system was maximized to 20 poses with an optimized exhaustiveness value. We performed the blind dock with multiple ligands per protein, and the run was repeated five times regarding the reproducibility of the test. The Vina output including the docking scores (Kcal/mol), upper and lower bound root mean square deviation (RMSD), and the number of hydrogen bonds and interacting residues are used to examine the best-docked conformations in the individual run.

### Interaction visualization

BIOVIA Discovery Studio Visualizer v21.1.0.20298 (BIOVIA, 2021) was used to visualize interacting residues between ligand and protein for structural analysis. The criteria of H-bonding and all other non-covalent interactions were set at the tool’s default parameters. The Python-based Molecular Viewer recorded the number of ligand modes and clusters that appeared on each protein–ligand docking system (Morris et al., 2009).

### Statistical analysis

Data collected in the study were analyzed and plotted with R version 4.1.3, and RStudio 2022.02.3 Build 461. Different packages of Tydiverse, Ggpubr, Ggpmisc, Corrr, Rstatix, FSA, and Rcompanion were used to create tidy data, data manipulation, publication-ready graphics, visualize and calculate correlation and regression model, perform basic statistical tests, and display compact letters of significant difference (Wickham et al., 2019; Kassambara, 2020a,b; Kuhn et al., 2020; Aphalo, 2021; Mangiafico, 2022; Ogle et al., 2022). Values were expressed as the mean and standard mean error using the Mann–Whitney, Kruskal–Wallis, and Dunn’s tests, with a *p*-value ≤0.05 for a significant difference.

## Results and discussion

### The higher the exhaustiveness value used, the better the docking score calculated and the fewer the variation of binding poses was

Exhaustiveness refers to the flexibility of atom conformations, so the optimization of exhaustiveness will give more favorable or consistent results in most cases (Forli et al., 2016). Specifically, it is vital that the optimization of exhaustiveness value was performed to evaluate the variation of binding poses. In this study, 3C^pro^ protein from 5C1U (later the proteins were called by its PBD ID) was selected for exhaustiveness screening due to its first discovered wild-type strain and having the crystallography model in complex with ligand. Meanwhile, the ligand Rupintrivir—an FDA-approved antiviral drug—was chosen due to its strong effect *in vitro* and advanced to clinical trials for *EV-A71* 3C^pro^ inhibition (Zephyr et al., 2021). The exhaustiveness in this experiment was primarily optimized exponentially by the default value of 8 to a maximum of 512.

**FIGURE 1:**
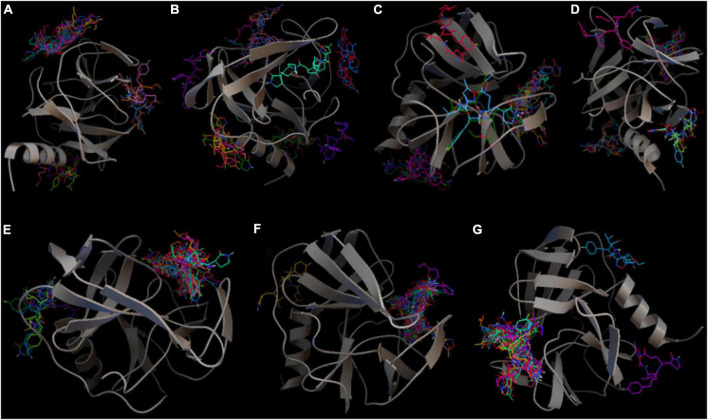
Clustering of models generated from the exhaustiveness optimization between ligand Rupintrivir and 3C^pro^ protein of 5C1U (PDB ID) with value **(A)** 8 (default), **(B)** 16, **(C)** 32, **(D)** 64, **(E)** 128, **(F)** 256, and **(G)** 512. The figure was illustrated by Python-based Molecular View with ribbon-shaped proteins and atom-shaped ligands.

In total, two essential points are needed to consider while assessing the molecular docking results of this exhaustiveness optimization: clusters and docking scores. First, a cluster is a predicted binding pocket on the target protein where multiple conformations of ligands bind to that protein (Kolluru et al., 2019). Python-based Molecular Viewer in the MGL tool package was used to visualize the clusters. Thus, the smaller number of clusters shows the more precision of the experiment, the more reliable in defining the binding site. As a result, the highest number of clusters was at the exhaustiveness 16 with 7 clusters, whereas the run of exhaustiveness values 8, 32, 64, and 512 displayed 3–4 clusters (Figure 1). Exhaustiveness 128 and 256 gave the best precision among all trials with 2 clusters, which would help to narrow the potential binding sites for better evaluation of the protein’s active site.

**FIGURE 2:**
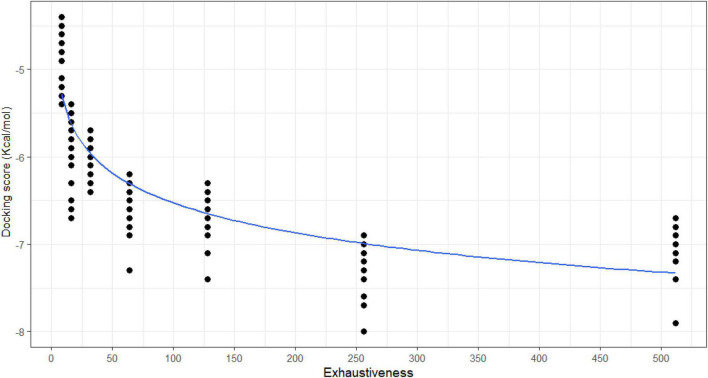
The correlation between exhaustiveness values and docking scores of each 20 binding poses of the system 5C1U—Rupintrivir. The correlation was calculated and drawn by RStudio followed logarithmic trendline with R correlation coefficiency 0.881 (*p*-value < 0.001).

The docking scores are the second criterion in exhaustiveness optimization, showing the difference in accessing the property of the ligand, which indicates how tightly Rupintrivir was bound to 5C1U. To put it another way, a smaller value indicates a stronger binding interaction, whereas a larger value illustrates a poor or non-existent connection. For analyzing which exhaustiveness gave the best docking scores, all 20 conformations of each exhaustiveness’s run were depicted in Figure 2 to observe the trendline. The first three-exhaustiveness values (8, 16, and 32) their docking scores were predicted to be no lower than −7.0 Kcal/mol, and the difference between the three binding modes was not significant. Regarding the higher exhaustiveness values, the first mode of each exhaustiveness was predicted to be in the range from −7.0 to −8.0 Kcal/mol. In contrast, the docking scores were calculated to be almost −8.0 Kcal/mol when the exhaustiveness rose from exhaustiveness 64. Even though the first mode of the exhaustiveness 512 gave the highest docking scores (docking scores of −7.9 Kcal/mol) compared to others, the magnitude of the docking scores is not significantly higher than the one of lower exhaustiveness value. The docking scores and exhaustiveness value in this experiment had the logarithmic correlation followed the equation *y* = −4.2611−0.49232ln *x* with a very significant value of *p*-value < 0.001.

Moreover, the exhaustiveness 256 resulted in two binding sites similar to exhaustiveness 128, with only one conformation binds differently from the other conformations in exhaustiveness 256. In addition, the docking scores of exhaustiveness 256 are much better and more consistent than exhaustiveness 128 (docking scores of −7.4 and −7.7 Kcal/mol, respectively). Consequently, the exhaustiveness 256 was picked as the optimized exhaustiveness value for further protein–ligand docking.

### Six potential binding sites were detected in *in silico* experiment on 21 systems of 5C1U and ligands

The 21 3C^pro^-inhibited ligands are divided into two groups based on their original sources: synthetic drugs (Rupintrivir and its derivatives, DC07090, GC376) and natural products (flavonoids). The molecular docking study was done using the Autodock Vina tool and BIOVIA Discovery Studio Visualizer version 4.5. The experiment was done five times for each system to collect 100 modes in total. In every 100 modes of each ligand–protein pair, the binding sites with more than 15 poses would be considered a popular cluster. This cluster was calculated with the mean and standard deviation and then categorized based on the interacting amino acid residues.

**FIGURE 3:**
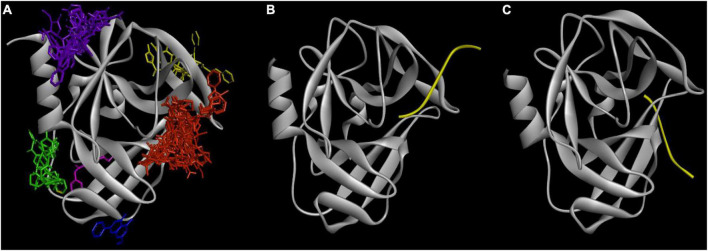
The consistency between *in silico* with *in vitro* experiments: **(A)** six popular clusters with different colors were made between 21 candidates with the 5C1U: red—cluster 1, green—cluster 2, purple—cluster 3, yellow—cluster 4, blue—cluster 5, and pink—cluster 6; and the crystallography model of **(B)** 3SJ9 and **(C)** 3SJK between 3C^pro^ protein (gray color) with its targeted peptide (yellow color) (Lu et al., 2011). The figure was illustrated by Python-based Molecular View with a ribbon-shaped proteins or polypeptide and atom-shaped ligands.

There are six binding sites in which 21 ligands bound to 5C1U (Figure 3A). Cluster 1 is the most favorable binding site where all 21 ligands interacted with 5C1U protein. Meanwhile, clusters 2–6 are observed in some ligand–protein systems. Table 2 displays the involved amino acid residues at different *in silico* binding sites making interactions with 21 ligands. Previously, Lu et al. (2011) and Wang et al. (2011) had reported that a Cys147-His40-Glu71 played as a catalytic triad in the active site, whereas Lu’s group study revealed that Tyr122, Phe124, Glu126, Leu125, Leu127, Ser128, Thr142, His161, Ile162, Gly164, and Phe170 amino acid residues marked the 3C^pro^ cleavage site. These reported interacting residues are similar to cluster 1, involving interactions from Arg39, His40, Glu71, Leu127, Ser128, Ala144, Gly145, Cys147, His161, Leu162, and Gly164 residues. This was proved by comparing side-by-side the formation of ligand–protein complexes in Figure 3. It is consistent in the cleavage position between cluster 1 and the polypeptide in 3C^pro^ (PDB ID: 3SJK and 3SJ9). Additionally, there is no significant difference in docking scores between clusters in a pair of ligands—5C1U system (Supplementary Material 2). This proves that despite being predicted to have multiple different binding sites, the docking scores between a ligand and 5C1U are stable, as well as always being high at cluster 1 rather than other clusters. Hence, cluster 1 here is confirmed to be portrayed as the active site of 3C^pro^ in our *in silico* experiment.

**TABLE 2:** Six clusters formed between 21 3C^pro^-inhibiting candidates and 5C1U with involved interacting residues at each cluster.

Binding site	Involved residues*
Cluster 1	Arg39, His40, Glu71, Leu127, Ser128, Ala144, Gly145, Cys147, His161, Leu162, Gly164
Cluster 2	Leu11, Arg12, Arg16, Glu50, His51
Cluster 3	Leu4, Ala7, Thr101, Phe113, Pro115, Thr152, Ser153, Val154
Cluster 4	Glu65, Gln66, Glu92, Ile94, His133, Arg134, Met136, Lys175
Cluster 5	Asp21, Trp48, Leu53
Cluster 6	Arg31, Arg33, Leu34, Val60, Leu74, Thr76

*The interacted residues were identified using the BIOVIA Discovery Studio Visualizer version v21.1.0.20298.

**FIGURE 4:**
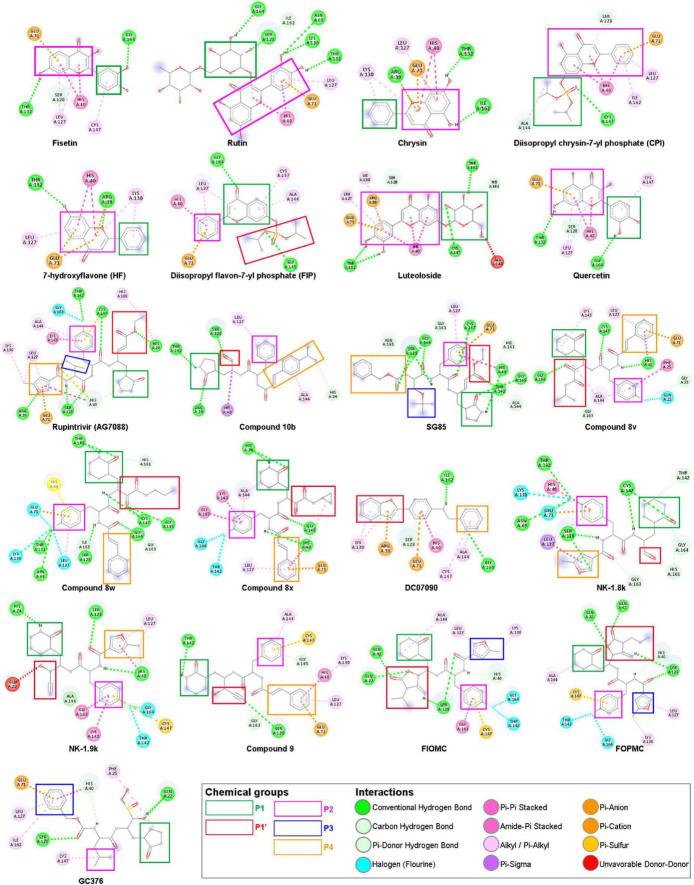
Chemical structures of 21 ligands with five interacted chemical groups—showed in colored rectangles—and their interacted amino acids—showed in colored circles—at cluster 1 with 5C1U. The figure was modified from the illustration of BIOVIA Discovery Studio Visualizer v21.1.0.20298 (BIOVIA, 2021).

Regarding the docking scores, several derivatives in the synthetic group had shown that they have better docking scores compared to Rupintrivir (Supplementary Material 2). We also found that the docking scores in the synthetic group were estimated to be better than the natural group flavonoids due to having more functional groups in their structures, as displayed in Figure 4. However, Rutin, classified as flavanols, gave the best docking scores when all 21 ligands docked against 5C1U (docking scores of −8.56 Kcal/mol). In the reported DC07090, Fisetin, Rutin, Chrysin, Diisopropyl chrysin-7-yl phosphate (CPI), and Luteoloside by the study of Ma et al. (2016) showed that DC07090 displayed excellent antiviral activities in cellular-based assay against 3C^pro^. Contrary to our *in silico* result, Rutin and CPI showed better inhibitory ability than DC07090. These differences in the antiviral inhibitory potency might be due to the different experimented *EV-A71* strains between *in vitro* and *in silico*, which were used in research, and the docking scores were calculated mathematically based on different interactions, which does not show the whole action of antiviral activity.

Looking in detail at the protein–ligand interaction in the next process, the binding pocket can be affected by five chemical groups corresponding to the Rupintrivir and its derivatives, where the essential features for ligand binding are presented (Figure 4 and Supplementary Material 3). The lactam group at position P1, mimicking a glutamine residue, formed a strong triple H-bond at the S1 subsite containing His161 at the bottom and Thr142 locates on one side wall of the pocket, which is consistent with the previous study (Lu et al., 2011). Thus, this P1 group drastically enhanced the inhibitory potency due to its ability to mimic the glutamine recognition site, which was highly conserved in the 3C cleavage sequence. Besides, the P1 group also interacted with Lys143, Ala144, Cys147, Leu162, Gly163, and Gly164 residues, according to the Wang’s group (Wang et al., 2011)**.** However, in our molecular docking study, the lactam group of Nk-1.9k and compound 10b bound to other amino acid residues such as His24 and Thr132 *via* hydrogen bonding. Meanwhile, the interaction between the glutamine surrogate ring of GC376 and 5C1U was *via* the π-alkyl bond of Phe25 residue, which is different from the research carried out by Kim et al. (2012). The P2 group as 4-fluorophenylalanine hydrophobically interacted with Leu127, Glu71, and His40. The negatively charged carboxylate side chain of Glu71 was revealed to be critical for preserving the overall design of the active site and maintaining the active conformation of His40 reported by Wang et al. (2011). Interestingly, the P2 group of most compounds in the synthetic group, except compound 8w, compound 9, Nk-1.8k, and SG85, is rotated to fit into the S1 pocket. This may be due to the non-available structures in the PubChem database, which were all manually re-drawn structures, or the proteins used for docking are from different sources with previous reports. Gly164 plays an essential role in the interaction of the P3—valine group, but this group mostly disappears in most derivatives because it points toward the solvent region (Lu et al., 2011). Similarly, the P3 group of GC376, which is a 6-membered aromatic ring, is confirmed to face toward the solvent by Kim et al. (2012), but our docking study shows that this group fits into the S2 pocket. The P4 functional group is usually a styryl or 5-methyl-3-isoxazole in most ligands of the synthetic group or even a Cbz group of SG85. Meanwhile, the S4 pocket can only allow tiny side chains, with alanine or glycine/valine preferred by the 3C^pro^ (Lu et al., 2011)**.** Therefore, these P4 functional groups moved into the S2 pocket instead. The S1′ subsite is a small leaving group-side pocket where Gly145 is the main interacting residue (Wang et al., 2017). However, the P1′ group of most ligands did not bind to Gly145. To be specific, α-keto amide of compound 8v interacted with Gly163 and Gly164; the cyanohydrin of Nk-1.9k and compound 9 interacted with Gln22 and Ser128, respectively. In addition, the formation of the unfavorable bonds between -OH groups and the amino acid residues affects the stability of ligands bound to proteins, despite obtaining high magnitudes of docking scores. However, the unfavorable bonds of these ligands are absent in other proteins, which can be lost during the interaction in the biological environment.

The α, β-unsaturated ester of Rupintrivir interacted with His24 and His108 whereas P1’s group of SG85 moved into the S1 pocket. The cyclized carbamates of FIOMC and FOPMC interacted with Gln22, Gln42, and Ser128. It is noticeable from Figure 4 that the oxazole group of DC07090 did not occupy with S1’ pocket, but it moved into the S2 pocket to form hydrophobic interactions with Arg39, Ser128, and Lys130 residues. Additionally, the pyridine ring of DC07090 fits into the S1 pocket instead of the S4 pocket, which is totally different from the Ma et al.’s (2016) report. These differences in DC0709 and GC376 could be due to the flexibility of the natural protein and its captured moment by X-ray crystallography. Although the model was taken from the same PDB ID, the energy minimization process could also produce minor differences that affected the interaction between ligand and protein. In addition, the docking tool used in the previous studies was not mentioned to explain the difference clearly. Regarding the natural group, the interactions mainly were exhibited by van der Waals contacts with the amino acid residues at S1 and S2 pockets. Besides, looking in detail at the structures of two natural compounds (Rutin and Luteoloside), which were estimated to have the best docking scores, Rutin has maltose in the side chain while glucose is attached to the Luteoloside’s side chain. Thus, we propose that the addition of the glucose group may help not only improve the docking scores but also increase the solubility of that natural compound. Although there is a difference between our interacted analysis and previous *in vitro* reports, it is easily explained due to the pose of the docking experiment. The *in silico* docking experiment was done with the highest docking scores based on the interacted calculation. This can lead to the same binding position, but different poses compared to *the in vitro* experiment. Thus, the study in interaction gave some differences in some ligands with protein.

### Mutation of glutamic acid to alanine at position 71 gives the significant impact on the binding activity of 21 3C^pro^-inhibiting candidates to the protein

**FIGURE 5:**
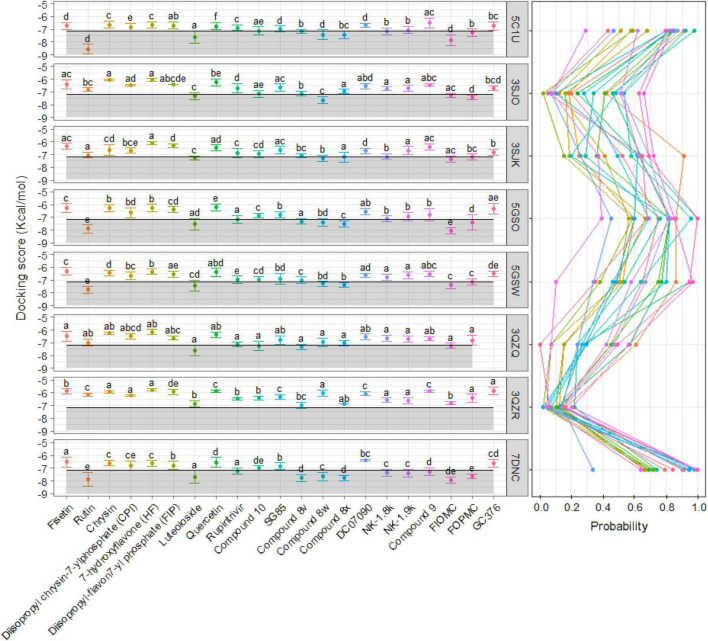
The average with standard deviation of docking scores **(left)** and the probability of models **(right)** at cluster 1—the active site—in each of 21 ligands and eight selected proteins. The shaded area was drawn based on the average docking score of all modal ligands at the active site with the 5C1U as a control. The significant difference was calculated by Kruskal–Wallis test and presented by different alphabets (α = 0.05) between each of eight proteins interacted with same ligand.

To find the effect of candidates on different strains or mutated proteins, a total of seven other 3C^pro^ proteins were docked against 21 previously selected inhibitors using Autodock Vina with the same experimental criteria. The average of all models bound at cluster 1 of 5C1U was calculated and used as the control value to compare the docking scores between ligands and proteins**.** Overall, the number of ligands that interacted at the active site of wild-type protein from strains of 5GSO, 3SJK, and 3SJO was not significantly different from the same ligands against 5C1U. Meanwhile, the interactions between the mutated proteins (PDB ID: 5GSW, 3QZQ, and 3QZR) and ligands decreased significantly or even had no interaction (Figure 5 and Supplementary Material 4). In terms of the docking scores of seven 3C^pro^ proteins, the average docking score of cloned protein (PDB ID: 7DNC) is better than or similar to the 5C1U. In detail, half of 21 ligands docked against cloned protein 7DNC to show better docking score compared to the interaction of 5C1U. To be specific, Rupintrivir showed improve inhibitory effect in other strains or mutations as follows: 7DNC > 5GSO = 3QZQ > 5C1U. Likewise, FIOMC and 8x also displayed good docking scores on 7DNC and 5GSO rather than 5C1U (Figure 5).

**FIGURE 6:**
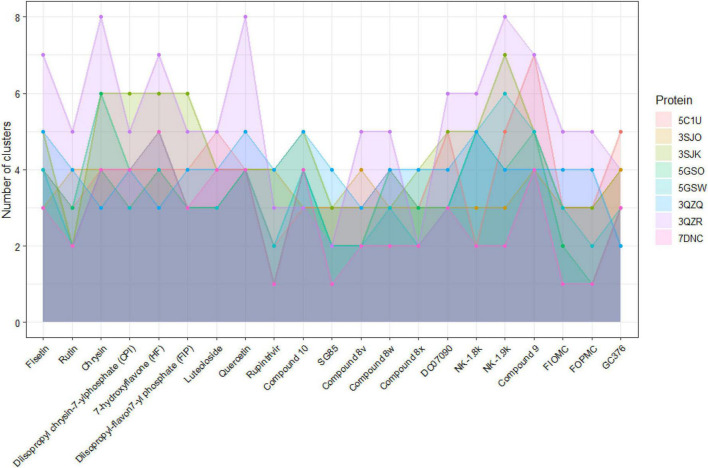
The number of clusters formed in docking each 21 ligands against each of eight proteins systems.

It is noticeable that the decline in docking scores occurred in mutated proteins. With the 3QZR, the docking scores not only become worse, but the ratio of the number of models interacting at the active site also drop sharply to less than 20% in all ligands (Figure 5); and the number of clusters predicted by Autodock Vina between 21 ligands and 3QZR is higher and more varied than other proteins (Figure 6). This shows that the mutation in 3QZR significantly affected the binding between candidates and the 3C^pro^. 3QZR consists of the 3C^pro^ protein having a mutation of strain BJ/CHN/2008 (PBD ID: 5GSO) from glutamic acid (E) to alanine (A) at position 71, which is belonged to the catalytic triad in the active-site region. This mutation hindered the binding of ligands on the active site with weaker interactions than the other protein. Meanwhile, the mutation with the same position from glutamic acid (E) through the amino acid of the same property—aspartic acid (D) in the 3QZQ did not have much difference in docking scores compared with the wild-type proteins as well as the number of clusters between twenty-one inhibitors and the target. Previous reports noted that His40 residue serves to prime the Cys147 side chain for nucleophilic attack on the scissile bond by abstracting a proton, whereas the role of the Glu71 is to provide electrostatic stabilization of the resulting positive charge on the His40. This catalytic triad (Cys147-His40-Glu71) plays a vital role in building a strong H-bond network, which is easily hydrolyzed by peptides or ligands (Wang et al., 2011). Besides, alanine is simply too short and has only one acidic group instead of two carboxyl groups as in glutamic acid, so it could not reach histidine. Thus, substituting glutamic acid with alanine resulted in having insufficient electron densities to stabilize the catalytic triad, whereas the substitution of aspartic acid could maintain part of the action due to the same property as glutamic acid. This also outcomes in the formation of hydrophobic interactions between His40 and ligands instead of H-bond, so the active site became less favorable toward ligands. As a result, the E71A has the biggest effect on binding positions due to the void of all proteolytic activity, whereas the E71D mutant was partially active. The other mutation in the BJ/CHN/2008 strain (PDB ID: 5GSW) is the substitution of an asparagine (Asn) to a serine (Ser) at the position 69 (N69S). According to Wang et al. (2017) studied the inhibitory effect of NK-1.8k, the report suggested that replacing asparagine with serine destabilized the S2 pocket, which negatively influenced the binding capability of ligands. This can be explained by Asn69 establishing a hydrogen bond with Glu71, which is important for the proteolysis event of *EV-A71* 3C^pro^. However, since the molecular structure of serine is shorter than asparagine, the hydrogen bond between Glu71 and Ser69 is disrupted (Wang et al., 2017). In contrast, the N69S mutation in our study has a minor effect on the number of binding sites as well as the docking scores. Besides, the number of clusters generated by the interaction between Rupintrivir, SG85, and 8x docked against eight proteins is less than other ligands. In addition, although FIOMC showed fewer clusters when docked against the cloned protein and its docking scores in all eight proteins are the lowest values, the number of clusters created in the other seven proteins is still much compared to other ligands.

Of the 21 ligands, FIOMC showed the strongest broad-spectrum anti-3C^pro^ activity. According to Xu et al. (2021), FIOMC had broad effects on five viral strains of 3C^pro^ with EC_50_s ranging from 80 to 125 nM *in vitro* cell-based experiments. Our docking studies have also shown the same trend that FIOMC can be used as a promising inhibitor for targeting multiple *EV-A71* 3C^pro^ proteins. The estimated docking scores were calculated and followed by 5GSO (−8.06 Kcal/mol) > 7DNC (−7.92 Kcal/mol) > 5C1U (−7.83 Kcal/mol) > 5GSW (−7.40 Kcal/mol) > 3SJK (−7.33 Kcal/mol) > 3SJO (−7.28 Kcal/mol) > 3QZQ (−7.23 Kcal/mol) > 3QZR (−6.82 Kcal/mol). As previously reported, although the EC_50_s of FIOMC were higher than Rupintrivir (Supplementary Material 1), we could not compare which ligand shows the best inhibitory effect due to the different materials in the two studies (Wang et al., 2011; Xu et al., 2021). Besides, we also evaluated the correlation between EC_50_/IC_50_ values and the docking scores of fifty ligands on the 5C1U protein were assessed and it did not follow the trend. This is easily understandable that the EC_50_/IC_50_ values were evaluated differently with even the same ligands and the viral strains carried out in the studies are also different with the ones in our docking study. This leads to the *in silico* docking method in this study to predict the ligand’s inhibitory ability toward the targeted proteins. Our study revealed that the docking scores of Rupintrivir against 3C^pro^ proteins are higher than that of FIOMC. This is due to the replacement of (S) γ-lactam ring by (S) δ-lactam ring in FIOMC could enhance the inhibitory potency of ligands against *EV-A71* 3C^pro^ (Zeng et al., 2016). In addition, the cyclized carbamate derivative replaces the α, β-unsaturated ester at the P1′ position of Rupintrivir, making the structure more stable. According to Wang et al. (2017) and Xu et al. (2021), the α, β -unsaturated ester is not favored in drug design because it is reported to build an irreversible covalent bond with the catalytic residues, resulting in many adverse effects in the treatment of virus infection. It is noticeable that the valine at the P3 position on Rupintrivir is reduced in FIOMC. The P3—the valine group—is positioned toward the solvent zone and makes contact with the Gly164 residue in Lu et al.’s (2011) work, so it does not affect the overall inhibitory capability. Thus, FIOMC promises to be a prospective antiviral candidate against wild-type and mutated 3C^pro^ proteins.

### Hesperidin would have broad antiviral spectrum on EV-A71 3C^pro^ as better than FIOMC

There were many different reports on the substances from natural resources that their strong inhibitions on *EV-A71 in vitro* have been proven previously (Arita et al., 2008; Tsai et al., 2011; Zhu et al., 2011; Ji et al., 2013; Li et al., 2015, 2017; Choi et al., 2016; Min et al., 2018; Dai et al., 2019; Yin et al., 2019). However, the mechanism of action was not known. These 29 ligands were collected and went through the same *in silico* experiment to investigate the possibility of 3C^pro^ inhibitors based on the result from the above experiment. To evaluate their 3C^pro^ inhibiting property, the percentage of docked modes bound at cluster 1 of 5C1U was compared with the minimum percentage of cluster-1 modes formed between 21 3C^pro^-inhibiting ligands and 5C1U previously. However, due to the missing information of some inhibitors on the PubChem database, most of the ligands were re-drawn, and the docking result would be partially affected by this. The 3D-sketched chemical structure will never show full potential as a crystallographic structure in a biological environment. The sketched one could be different with different tools or people. Several factors need to be considered, namely, angles between bonds, interactions among covalent bonds, total energy, and different types of bonds (e.g., plain bonds, wedged bonds, and dashed bonds) in three-dimensional space, which are impossible to manipulate. Moreover, after any modification, the structural optimization had been done giving various 3D structures despite having similar outside resemblance. 0.43 was chosen as the threshold from the probability of models at cluster 1 formed between 5C1U and ten crystallized, DC07090, Fisetin, Rutin, Chrysin, CPI, Luteoloside, Quercetin, 7-hydroxyflavone, Diisopropyl-flavon7-yl phosphate, and GC376. So, any ligand in this 29 ligands’ group with the percentage of docked modes at cluster 1 with 5C1U higher or equaled 0.43 was proposed as having 3C^pro^ inhibited property. The percentage of active site-bound modes with other proteins is not the main criteria for the elimination due to their highly affected different amino acids or mutations as the previous results, which gave the false-negative evaluation. The second criteria to conclude on the 3C^pro^-inhibiting action of the ligand are the docking scores formed between the ligand and protein. Although the ligand possesses a high probability of binding at the active site of 3C^pro^, if it had a docking score higher than −7.083 Kcal/mol, the inhibition is considered as low for 3C^pro^.

**FIGURE 7:**
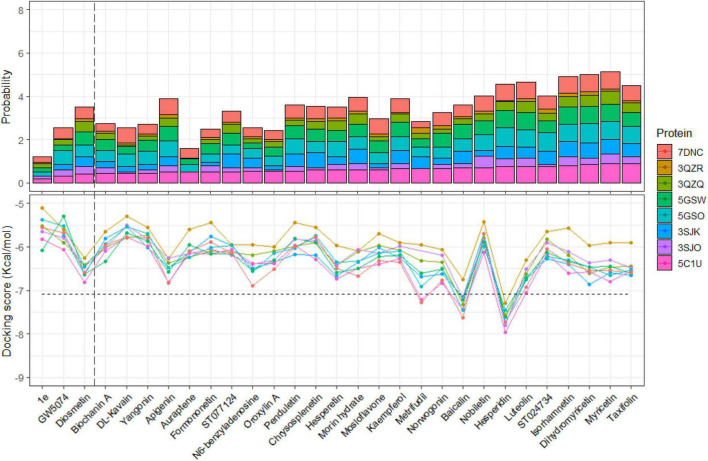
Percentage **(upper)** and average of docking scores **(lower)** of models bound at active site of each 29 ligands to eight different proteins. The vertical and horizontal dashed lines are the thresholds of the probability and the average of docking scores, respectively.

As a result, shown in Figure 7, we can propose that the substances are as follows: Metrifudil, Baicalin and Hesperidin could have inhibiting properties on the 3C^pro^. Their action on different proteins also had the same influence as the previous twenty-one inhibited ligands as the percentage of active site-bound modes was reduced in 3QZQ and 3ZQR. It can be easily seen that most of the substances with a low percentage of modes at cluster 1 also had low docking scores on 5C1U protein. These results show the consistency in both two evaluating criteria of docking score and probability of active-site modes, especially in proposed non-specific 3C^pro^ ligands. The molecular docking study revealed that the docking scores of Hesperidin were estimated to be the best in all eight 3C^pro^ proteins, and it was even better than FIOMC or Rupintrivir. Using the SwissADME web server, the MlogP of each ligand was accessed to evaluate their octanol–water partition coefficient, which is a measure of how hydrophilic or hydrophobic a molecule is (Supplementary Material 1). The MlogP value is a straightforward, yet effective structure-based method developed by Moriguchi’s team for estimating logP values, and the MlogP threshold should be less than or equal to 4.15 (Lipinski et al., 2012; Daina et al., 2017). Due to the presence of more hydroxyl groups, Hesperidin has lower MlogP than FIOMC and Rupintrivir. This may affect the permeability across the lipid membrane; however, a supporting carrier can be considered to assist the ligand passing through the membrane if it is more beneficial. Besides the mandatory properties to become a potent drug candidate, natural products, such as flavonoids, are known to have multi-targets by not only targeting the virus, but also the host cell. For example, it was found that Tylophorine derived from *Tylophora indica* targets the common pro-inflammatory activities of host cells to viral SARS-CoV-2 infections such as the viral RNA replication, cellular JAK2, and media-dominant NF-B activation (Al-Harrasi et al., 2022). Therefore, evaluating polypharmacology is a necessity to screen potential candidates for effective metabolism and activities; and the Lipinski Rule of Five is one of the criteria to assess the polypharmacology properties of inhibitors. The assessment of inhibitors on various biological targets in the CYP family, an enzyme in the host cell, is one among these criteria. However, we do not mention this in our study. Aside, the ratio of the number of modes at the active sites also ranked high compared to other ligands. The docking scores and the ratio of modes at the active site of both wild-type and mutations witnessed a decrease, especially the lowest value observed in the interaction with 3QZR, consistent with previous cases of twenty ligands. This proved that mutation has a big impact on the number of binding sites as well as the docking score value.

**FIGURE 8:**
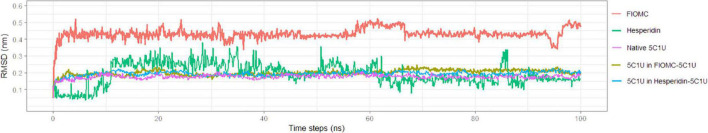
The stability of protein 5C1U and ligands through molecular dynamics simulation of two systems 5C1U-FIOMC and 5C1U-Hesperidin. The MD simulation was done with Gromacs version 2022.2 through 100 ns (Berendsen et al., 1995; van der Spoel et al., 2005; Abraham et al., 2015).

The molecular dynamic (MD) simulation had been carried out through (100 ns) using basic settings to investigate the stability of the apo-form of protein and the docked complexes and to acquire more insight on two systems of the 5C1U-Hespirindin and 5C1U-FIOMC (Supplementary Material 5). A fundamental measurement for assessing the variations in structural stability through the simulated times in docked complexes and the native protein form is the RMSD, which is determined independently for the protein and ligands (Figure 8; Bouback et al., 2021). The RMSD values in all five components are less than 5Å, according to the RMSD plot in Figure 8 with regard to the original structure for the duration of the MD simulation. Except for a drop in the FIOMC-5C1U complex at the beginning that results from the relaxation of the structures, both free and complex models in the RMSD plots exhibit a jump from the time of 0.2 ns. Overall, the average RMSD of protein 3C^pro^ in two systems is about 0.2 nm (2Å), which means the stability of the protein through the MD simulation. Meanwhile, the RMSD of ligands was different through 100-ns simulated time. Hesperidin’s RMSD shows the transition of ligand from time of 8-ns with the average around 0.2 nm. The most difference was observed at 11–40 ns. However, this 5C1U-Hespirindin is still more stable than the system 5C1U-FIOMC. The change in distance between the simulated ligand with the original one was recorded at more than 0.4 nm through 100-ns simulation. Further MD study on other systems could show the dynamic interaction. Therefore, Hesperidin should be researched more about its interaction *in silico* and *in vitro* experiments in the future. These 29 ligands were also represented as a mini database for the further experiment *in silico* screening active compounds for 3C^pro^ inhibitors. Moreover, the result showed a good agreement in using docking scores and percentage of binding site modes to make a conclusion on potential ligands.

## Conclusion

Currently, no therapies can successfully prevent the occurrence and mutations of *EV-A71*, with symptomatic and supportive treatment being the only options. Despite the fact that *in vitro* assays are now being used to investigate inhibitors targeting 3C^pro^, one of the constraints is the limited number of ligands and 3C^pro^ strains available in the research. Besides, viruses frequently mutate, and random mutations can change protein–drug interactions, so *in silico* screening will be faster, more economical, and timelier if new mutations occur. This not only leads to separate the inconsistent studies but also challenges in finding the good inhibitors against the 3C^pro^. Moreover, evaluation of protein–ligand complexes by molecular docking is a time-saving and money-saving method, which aids in faster identifying the potency of ligands toward the biological targets by ranking with the scoring function. Another advantage is the use of these tested ligands in our study that could be used as controls for later screening bioactive or synthetic drugs faster. However, there are two biggest drawbacks of this method including the manual clustering of ligand conformations with more than 100 ligands and the results of docking. When assessing whether an inhibitor has the ability to suppress a protein, the ratio of ligand-bound at the same active site, as well as the binding affinity value, is critical. Hence, enhancing Autodock Vina’s clustering algorithm is necessary to screen a huge database. The latter issue—the scoring function—is the estimation of the free energy of interaction between the protein and ligand rather than computing it. Thus, thanks to the rapid increase in computational capabilities, the future of scoring function development should focus on how to enhance the accuracy of the docking scores. Regarding our *in silico* study, we first determined the exhaustiveness value of 256, which gave the least variation in binding sites and good docking scores as well. Then, the key interacting residues at the active binding site between the interactions of twenty-one reported ligands and eight 3C^pro^ proteins were identified as references for other strains or mutations of *EV-A71* 3C^pro^ in the future. Of 21 ligands, FIOMC was determined as having a broad anti-3C^pro^ spectrum from our docking results. In total, 29 new natural compounds are used and considered as a miniature database for protein inhibitors screening, evaluating important criteria in screening which is supportive in widening the database. Our study also revealed that Hesperidin—a flavanone—showed a potential inhibition in *EV-A71* 3C^pro^, and its docking scores are even better than FIOMC, but the *in vitro* study is limited. Therefore, future *in vitro* experimental work on inhibiting 3C^pro^ activity of Hesperidin should be performed to compare with FIOMC’s *in vitro* result as well as validate the current *in silico* study.

## Data availability statement

The original contributions presented in this study are included in the article/Supplementary material, further inquiries can be directed to the corresponding author.

## Author contributions

TTVL and P-CD carried out the study as well as drafted and modified the manuscript. Both authors read and approved the final manuscript.
