# Supplementary material for: Molecular docking study of various Enterovirus—A71 3C protease proteins and their potential inhibitors
Source: Front Microbiol. 2022 Sep 29;13:987801. doi: 10.3389/fmicb.2022.987801 (PMC9563145; doi:10.3389/fmicb.2022.987801)
Supplement: Supplementary file 1 [file Data_Sheet_1.pdf]

Supplementary S01. Phytochemicals and their derivatives as drug-candidates for treating Hand, Foot, and Mouth disease.

No	Compound name	Chemical structures	IC_{50}/ EC_{50} (μM)			MlogP ** calculated by the SwissADME webserver*	Group
			IC_{50} (dose-dependent manner)	IC_{50} in vitro	EC_{50}		
1	Luteoloside (Cao et al., 2016)	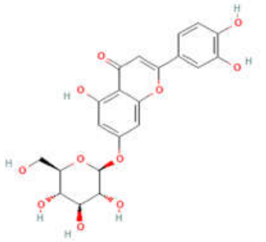	360		430	-2.1	
2	Quercetin (Yao et al., 2018)	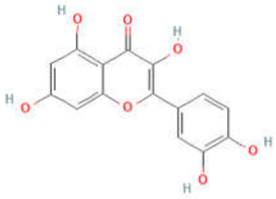	12.1	8.8	1.2	-0.56	
3	Chrysin (Wang et al., 2014b)	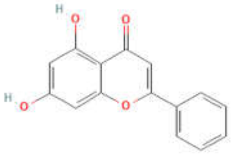	24.12	4.03	15.89	1.08	$3C^{pro}$ inhibitors
4	Diisopropyl chrysin-7-yl phosphate (CPI) (Wang et al., 2014b)	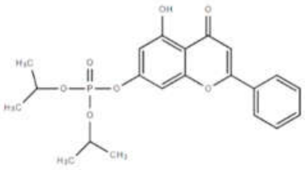	13.86	1.9	9.06	1.6	
5	7-hydroxyflavone (HF) (Wang et al., 2014a)	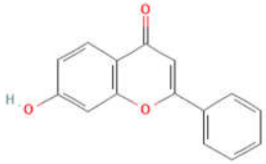	23.45		19.95	1.66	

6	Diisopropyl-flavon7-yl phosphate (FIP) (Wang et al., 2014a)	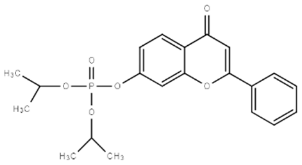	13.63	2.48	9.87	2.13	
7	Fisetin (Lin et al., 2012)	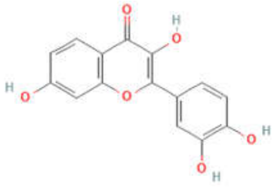	84.48	142.8		-0.03	
8	Rutin (Lin et al., 2012)	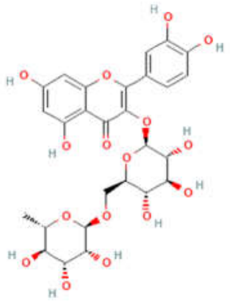	109.63	83		-3.89	$3C^{pro}$ inhibitors
9	AG7088 (Wang et al., 2011)	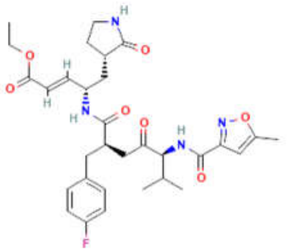		2.3	0.001	1.51	
10	Compound 10b (Kuo et al., 2008)	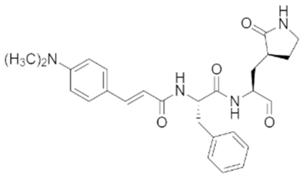		< 0.5	0.018	1.02	
11	NK-1.8k (Wang et al., 2017)	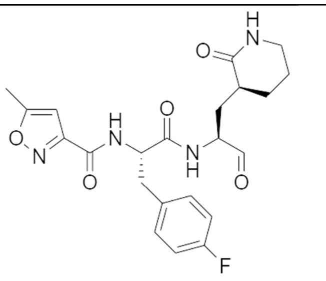		0.11	0.108	0.68	

12	NK-1.9k (Wang et al., 2017)	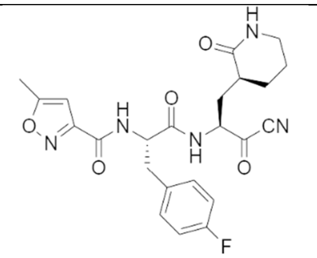		0.31	0.037	0.02	
13	Compound 9 (Wang et al., 2017)	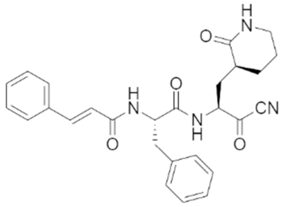		1.13	0.12	0.83	
14	SG85 (Tan et al., 2013)	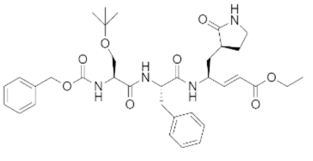			1	1.23	
15	FOPMC (Xu et al., 2021)	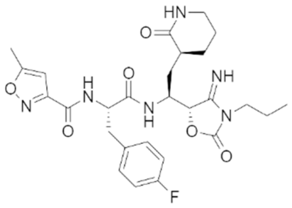			0.21	1.27	$3C^{pro}$ inhibitors
16	FIOMC (Xu et al., 2021)	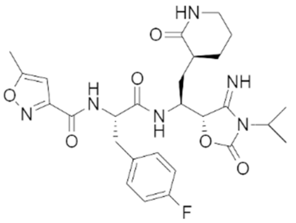			0.1	1.27	
17	8v (Zeng et al., 2016)	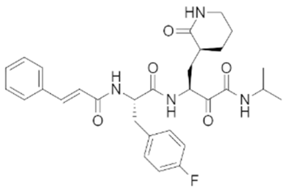		1.32	1.12	1.33	
18	8w (Zeng et al., 2016)	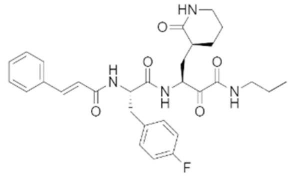		1.88	1.08	1.33	
19	8x (Zeng et al., 2016)	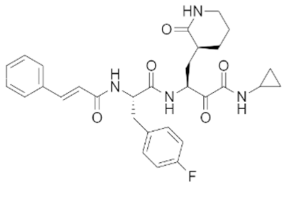		1.52	1.55	1.33	

20	DC07090 (Ma et al., 2016)	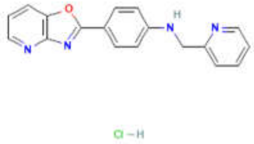		21.72	22.09	2.41	$3C^{pro}$ inhibitors
21	Apigenin (Dai et al., 2019)	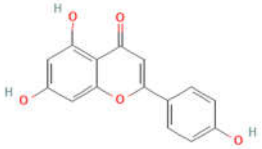			24.74	0.52	
22	Luteolin (Dai et al., 2019)	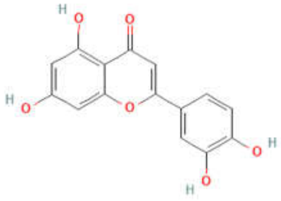			13.5	-0.03	
23	Kaempferol (Dai et al., 2019)	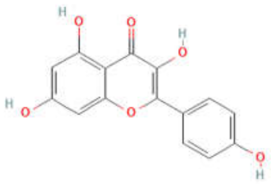			52.75	-0.03	*EV-A71* inhibitors
24	Formononetin (Dai et al., 2019)	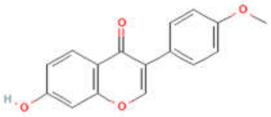			12.5	1.33	
25	Penduletin (Dai et al., 2019)	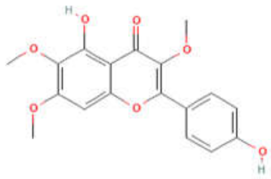			0.63	0.17	

26	Isorhamnetin (Dai et al., 2019)	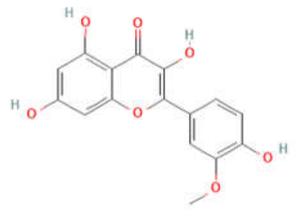			60.7	-0.31	
27	Biochanin A (Li et al., 2017)	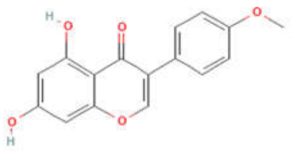				0.77	
28	Hesperetin (Tsai et al., 2011)	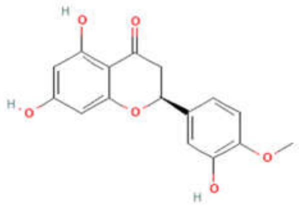				0.41	*EV-A71* inhibitors
29	Hesperidin (Tsai et al., 2011)	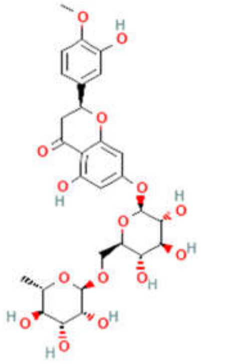				-3.04	
30	Nobiletin (Yin et al., 2019)	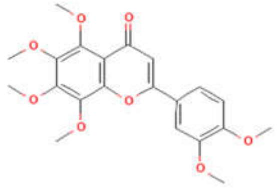				0.34	

31	Morin hydrate (Yin et al., 2019)	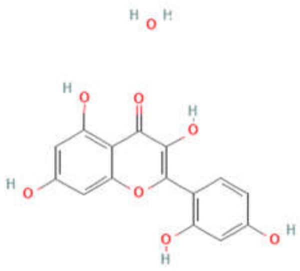				-0.56	
32	Taxifolin (Yin et al., 2019)	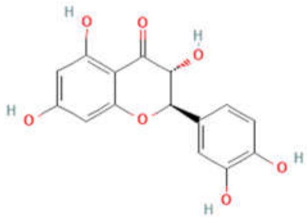				-0.64	
33	Diosmetin (Yin et al., 2019)	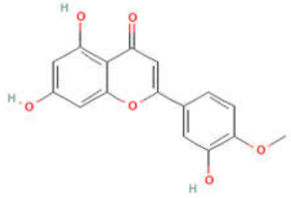				0.22	*EV-A71* inhibitors
34	Dihydromyricetin (Yin et al., 2019)	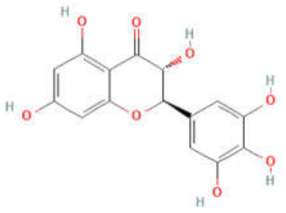				-1.16	
35	Chrysosplenetin (Zhu et al., 2011)	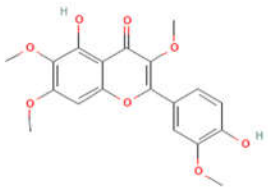		0.20		-0.12	

36	ST077124 (Min et al., 2018)	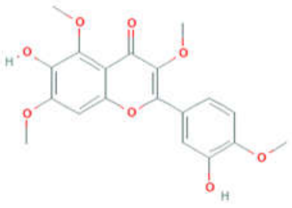				-0.12	
37	ST024734 (Min et al., 2018)	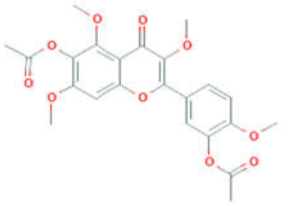				0.7	
38	Norwogonin (Choi et al., 2016)	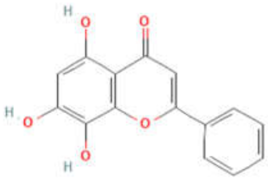		31.83		0.52	*EV-A71* inhibitors
39	Oroxylin A (Choi et al., 2016)	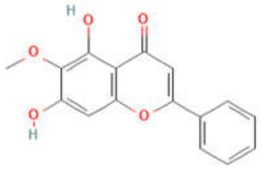		14.91		0.77	
40	Mosloflavone (Choi et al., 2016)	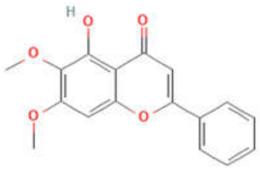		37.72		1.01	

41	Baicalin (Li et al., 2015)	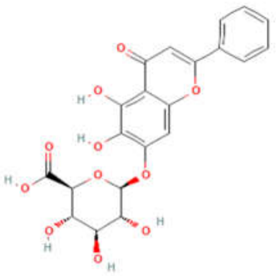	4.96			-1.63	
42	Yangonin (Li et al., 2017)	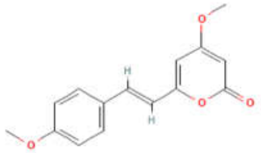			12.83	1.74	
43	DL- Kavain (Li et al., 2017)	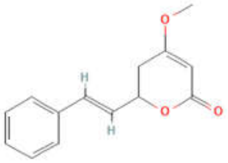				1.95	*EV-A71* inhibitors
44	Metrifudil (Arita et al., 2008)	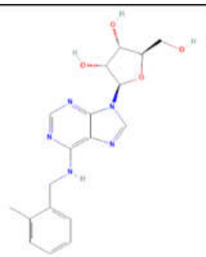		1.3		-0.87	
45	N^6-benzyladenosine (Arita et al., 2008)	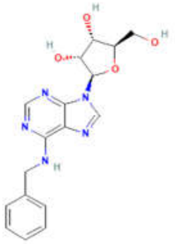		0.10		-1.1	
46	GW5074 (Arita et al., 2008)	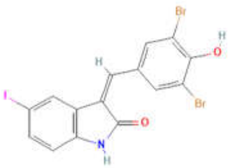		2.0		4.18	

47	1e (Ji et al., 2013)	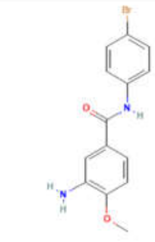		5.7 - 12		2.73	
48	Auraptene (Dai et al., 2019)	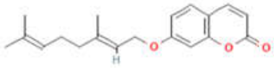			3.19	3.51	*EV-A71* inhibitors
49	Myricetin (Yin et al., 2019)	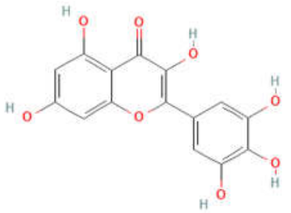				-1.08	
50	GC376 (Kim et al., 2012)	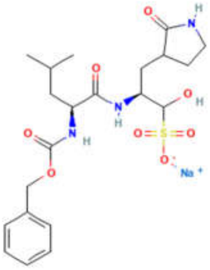		10.3 ±2.4		-0.08	
