# Supplementary material for: Molecular docking study of various Enterovirus—A71 3C protease proteins and their potential inhibitors
Source: Front Microbiol. 2022 Sep 29;13:987801. doi: 10.3389/fmicb.2022.987801 (PMC9563145; doi:10.3389/fmicb.2022.987801)
Supplement: Supplementary file 2 [file Data_Sheet_2.pdf]

Supplementary S02: Average and standard deviation of docking score (Kcal/mol) in each popular cluster formed in docked 5C1U-ligand systems

Ligand	Binding site					
	**Cluster 1**	**Cluster 2**	**Cluster 3**	**Cluster 4**	**Cluster 5**	**Cluster 6**
Fisetin	-6.748±0.266[a]	-	-	-	-	-
Rutin	-8.558±0.377[a]	-	-	-	-	-
Chrysin	-6.637±0.293[a]	-	-6.439±0.114[b]	-	-6.460±0.206[ab]	-
CPI	-6.824±0.304[a]	-	-6.727±0.241[a]	-	-	-
HF	-6.647±0.240[a]	-	-	-	-	-
FIP	-6.698±0.280[a]	-	-6.431±0.227[b]	-	-	-
Luteoloside	-7.606±0.474[a]	-	-	-	-	-
Quercetin	-6.758±0.294[a]	-	-	-	-	-
Rupintrivir	-6.880±0.234	-	-	-	-	-
Compound 10	-7.134±0.332[a]	-	-	-	-	-
SG85	-6.986±0.234[a]	-	-	-	-	-
Compound 8v	-7.167±0.171[a]	-	-	-	-	-
Compound 8w	-7.416±0.368[a]	-	-7.227±0.276[a]	-	-	-
Compound 8x	-7.431±0.292[a]	-	-7.456±0.268[a]	-	-	-
DC08090	-6.794±0.165[b]	-6.689±0.151[ab]	-	-	-	-6.594±0.106[a]
NK-1.8k	-7.171±0.260	-	-	-	-	-
NK-1.9k	-7.056±0.263[a]	-	-6.872±0.127[b]	-6.856±0.175[b]	-	-
Compound 9	-6.507±0.403[b]	-6.353±0.365[b]	-6.978±0.401[a]	-6.353±0.197[b]	-	-
FIOMC	-7.846±0.412[a]	-	-	-	-	-
FOPMC	-7.272±0.294[a]	-	-6.993±0.149[b]	-	-	-
GC376	-6.733±0.335[a]	-6.535±0.203[b]	-6.677±0.220[ab]	-6.420±0.045[ab]	-	-6.817±0.160[ab]

**Different alphabets show the difference in mean between subgroups in each ligand-protein system. The analysis was done by Mann-Whitney test (2 samples) or Kruskal-Wallis tests (> 2 samples) with significant level at 0.05 by R studio.*

***NA: the standard deviation is not available.*

****(-): the cluster is not available.*
