# Supplementary material for: Molecular docking study of various Enterovirus—A71 3C protease proteins and their potential inhibitors
Source: Front Microbiol. 2022 Sep 29;13:987801. doi: 10.3389/fmicb.2022.987801 (PMC9563145; doi:10.3389/fmicb.2022.987801)
Supplement: Supplementary file 3 [file Data_Sheet_3.pdf]

Supplementary S03: Hydrogen bonds at the cluster 1 formed between the best model of each ligand with the 5C1U protein.

Ligand	Interacted amino acid	Distance (Å)
Fisetin	Thr132	2.86
	Gly164	2.99
Rutin	Asn69	3.31
	Ser128	2.71 (O) – 3.91 (ring)
	Lys130	2.07
	Thr132	2.02
	Ile162	3.75
	Gly164	2.50
Chrysin	Arg39	3.18
	Thr132	1.88
	Ile162	1.96
CPI	Ser128	3.64
	Ala144	3.34
	Cys147	3.61
HF	Arg39	3.23
	Thr132	2.69
FIP	Gly145	2.93
	Gly164	3.19
Luteoloside	Ser128	3.89
	Thr132	2.08 – 2.78
	Thr142	2.29 – 2.70
	Cys147	3.57
	His161	3.76
Quercetin	Ser128	3.59
	Thr132	2.66
	Gly164	2.5
Rupintrivir	His24	3.13
	Arg39	3.12
	His40	3.53
	Ser128	2.04 (H) – 2.70 (O)
	Thr142	2.80 – 3.04
	Cys147	3.70
	Gly163	3.46
Compound 10	His24	3.77
	Arg39	2.55
	Ser128	2.21
	Thr132	2.37

Ligand	Interacted amino acid	Distance (Å)
SG85	His40	2.81
	Ser128	2.21 (H) – 2.98 (O)
	Thr142	3.11
	Ala144	3.45
	Gly145	3.15
	Cys147	3.51
	His161	3.54
	Gly163	3.38
	Gly164	2.34 (H) – 3.08 (O)
	Asn165	3.98
Compound 8v	Gln22	3.34
	Gly23	3.57
	His40	3.27
	Cys147	3.41
	Gly163	3.5
	Gly164	2.81
Compound 8w	Asn69	3.61
	Glu71	3.16
	Leu127	3.67
	Ser128	1.93
	Lys130	3.611
	Thr132	3.46
	Thr142	2.08 (H) – 3.13 (O)
	Gly145	3.06
	Cys147	2.95 (H) – 3.29 (O)
	His161	3.37
	Ile162	1.93
	Gly163	3.46
	Gly164	3.15
Compound 8x	His24	2.44 (H) – 3.20 (CO)
	His40	2.56
	Thr142	3.59
	Gly145	3.03
	Gly164	3.67
DC08090	Ser128	3.54 (ring) – 3.67 (O)
	Ile162	2.20
	Gly164	3.16
NK-1.8k	Asn69	3.62
	Glu71	3.18
	Ser128	1.8 (H) – 2.91 (N)
	Lys130	3.57
	Thr132	3.11
	Thr142	3.41
	Cys147	2.96 (H) – 3.56 (O)
	His161	3.68
	Gly163	3.32
	Gly164	3.62

Ligand	Interacted amino acid	Distance (Å)
NK-1.9k	His24	2.37
	His40	2.6
	Ser128	3.12 (O) – 3.6 (ring)
	Thr142	3.15
	Gly164	3.29
Compound 9	Ser128	3.12
	Thr142	2.31
	Gly145	3.56
	Gly163	3.78
FIOMC	Gln22	3.4
	His30	2.81
	Gln42	3.11
	Ser128	2.39 (H) – 3.04 (O)
	Thr142	3.21
	Gly164	3.34
FOPMC	Gln22	2.95
	His30	2.75
	Gln42	3.24
	Ser128	2.73 (H) – 3.14 (O)
	Thr142	3.47
	Gly164	3.24
GC376	Gln22	2.02 (H) – 3.01 (O)
	His40	3.03
	Ser128	2.82
