# Supplementary material for: Molecular docking study of various Enterovirus—A71 3C protease proteins and their potential inhibitors
Source: Front Microbiol. 2022 Sep 29;13:987801. doi: 10.3389/fmicb.2022.987801 (PMC9563145; doi:10.3389/fmicb.2022.987801)
Supplement: Supplementary file 4 [file Data_Sheet_4.pdf]

Supplementary S04. The binding sites formed between each twenty-one 3C^{pro}-inhibited ligands with different proteins were shown in average and standard deviation of docking score (Kcal/mol) in cluster 1 and check mark in other clusters.

**Protein**	**Ligand**	**Binding site**					
		**Cluster 1**	**Cluster 2**	**Cluster 3**	**Cluster 4**	**Cluster 5**	**Cluster 6**
	Fisetin	-6.410±0.351					✓
	Rutin	-6.807±0.126			✓		
	Chrysin	-6.056±0.078	✓				✓
	CPI	-6.467±0.049	✓				✓
	HF	-6.062±0.115	✓				✓
	FIP	-6.400±0.000	✓				✓
	Luteoloside	-7.332±0.257	✓				✓
	Quercetin	-6.258±0.264					✓
	Rupintrivir	-6.696±0.350					✓
	Compound 10	-7.159±0.286					✓
3SJO	SG85	-6.655±0.280	✓				
	Compound 8v	-7.138±0.175	✓				✓
	Compound 8w	-7.650±0.271	✓				✓
	Compound 8x	-6.950±0.151	✓				✓
	DC08090	-6.557±0.199					✓
	NK-1.8k	-6.763±0.148	✓				✓
	NK-1.9k	-6.714±0.222					✓
	Compound 9	-6.450±0.107	✓				✓
	FIOMC	-7.274±0.167					✓
	FOPMC	-7.410±0.179					✓
	GC376	-6.700±0.173	✓				✓
	Fisetin	-6.346±0.220	✓		✓		
	Rutin	-7.043±0.219					
	Chrysin	-6.616±0.417	✓	✓			
	CPI	-6.680±0.193	✓				✓
	HF	-6.089±0.133	✓				✓
	FIP	-6.300±0.141	✓				✓
	Luteoloside	-7.261±0.161	✓				✓
	Quercetin	-6.468±0.227			✓		
	Rupintrivir	-6.878±0.350	✓				
	Compound 10	-6.952±0.265					✓
3SJK	SG85	-6.641±0.273	✓				
	Compound 8v	-7.060±0.147	✓				✓
	Compound 8w	-7.267±0.238	✓				✓
	Compound 8x	-7.194±0.394	✓				✓
	DC08090	-6.712±0.195	✓				✓
	NK-1.8k	-7.151±0.215					✓
	NK-1.9k	-6.679±0.323					
	Compound 9	-6.405±0.262	✓				✓
	FIOMC	-7.342±0.240	✓				
	FOPMC	-7.178±0.217	✓				
	GC376	-6.797±0.239	✓				

Protein	Ligand	Binding site					
		Cluster 1	Cluster 2	Cluster 3	Cluster 4	Cluster 5	Cluster 6
	Fisetin	-6.264±0.329					
	Rutin	-7.905±0.306					
	Chrysin	-6.287±0.286					
	CPI	-6.632±0.397	✓				
	HF	-6.267±0.289	✓				
	FIP	-6.405±0.233	✓				
	Luteoloside	-7.529±0.449					
	Quercetin	-6.223±0.279					
	Rupintrivir	-7.142±0.298					
	Compound 10	-6.862±0.206					✓
5GSO	SG85	-6.807±0.243					
	Compound 8v	-7.320±0.215	✓				
	Compound 8w	-7.391±0.279	✓				
	Compound 8x	-7.510±0.235	✓				
	DC08090	-6.569±0.258					✓
	NK-1.8k	-7.121±0.243					
	NK-1.9k	-6.940±0.313					
	Compound 9	-6.808±0.497	✓				
	FIOMC	-8.062±0.264	✓				
	FOPMC	-7.406±0.620					
	GC376	-6.302±0.413	✓				
	Fisetin	-6.300±0.316	✓				
	Rutin	-7.760±0.258					
	Chrysin	-6.429±0.209	✓				✓
	CPI	-6.666±0.295	✓				
	HF	-6.350±0.192	✓				
	FIP	-6.553±0.246	✓				✓
	Luteoloside	-7.454±0.428					
	Quercetin	-6.394±0.305					
	Rupintrivir	-6.995±0.299					
	Compound 10	-6.940±0.204					✓
5GSW	SG85	-6.894±0.429	✓				
	Compound 8v	-7.001±0.244	✓				
	Compound 8w	-7.293±0.188	✓				
	Compound 8x	-7.373±0.195	✓				
	DC08090	-6.635±0.169					✓
	NK-1.8k	-6.787±0.267					
	NK-1.9k	-6.636±0.270	✓				
	Compound 9	-6.530±0.221	✓				✓
	FIOMC	-7.397±0.259					
	FOPMC	-7.156±0.239					
	GC376	-6.503±0.206	✓		✓		✓

**Protein**	**Ligand**	**Binding site**					
		**Cluster 1**	**Cluster 2**	**Cluster 3**	**Cluster 4**	**Cluster 5**	**Cluster 6**
3QZQ	Fisetin	-6.487±0.391	✓	✓			
	Rutin	-6.987±0.274	✓				
	Chrysin	-6.247±0.092	✓	✓			
	CPI	-6.500±0.208	✓	✓			
	HF	-6.160±0.206	✓	✓			
	FIP	-6.629±0.160	✓	✓			
	Luteoloside	-7.589±0.411	✓				
	Quercetin	-6.372±0.209					✓
	Rupintrivir	-7.122±0.197	✓	✓			
	Compound 10	-7.250±0.354	✓	✓			
	SG85	-6.793±0.338	✓				
	Compound 8v	-7.321±0.216	✓	✓			
	Compound 8w	-6.958±0.328	✓	✓			
	Compound 8x	-7.041±0.232	✓	✓			
	DC08090	-6.529±0.216	✓	✓			
	NK-1.8k	-6.647±0.236		✓			
	NK-1.9k	-6.700±0.225		✓			
	Compound 9	-6.686±0.168	✓	✓			
	FIOMC	-7.237±0.210		✓			
	FOPMC	-6.818±0.397		✓			
	GC376		✓	✓			
3QZR	Fisetin	-5.891±0.181			✓		✓
	Rutin	-6.169±0.120	✓				✓
	Chrysin	-5.950±0.109	✓			✓	✓
	CPI	-6.225±0.050	✓		✓	✓	
	HF	-5.780±0.103	✓		✓		✓
	FIP	-5.950±0.212	✓		✓		✓
	Luteoloside	-6.877±0.255	✓	✓			✓
	Quercetin	-5.882±0.087			✓		✓
	Rupintrivir	-6.477±0.073	✓				
	Compound 10	-6.427±0.164	✓				✓
	SG85	-6.340±0.195	✓				
	Compound 8v	-6.986±0.254	✓				✓
	Compound 8w	-6.050±0.238	✓				
	Compound 8x	-6.900±0.000	✓				
	DC08090	-6.071±0.149	✓			✓	✓
	NK-1.8k	-6.593±0.177	✓				
	NK-1.9k	-6.621±0.236	✓		✓		
	Compound 9	-5.875±0.096	✓				✓
	FIOMC	-6.820±0.132	✓				
	FOPMC	-6.427±0.313	✓				
	GC376	-5.850±0.295	✓		✓		✓

Protein	Ligand	Binding site					
		Cluster 1	Cluster 2	Cluster 3	Cluster 4	Cluster 5	Cluster 6
	Fisetin	-6.518±0.388					✓
	Rutin	-7.860±0.529					
	Chrysin	-6.602±0.220					
	CPI	-6.813±0.378					
	HF	-6.622±0.214					✓
	FIP	-6.784±0.316					✓
	Luteoloside	-7.679±0.503					
	Quercetin	-6.538±0.379					✓
	Rupintrivir	-7.213±0.229					
	Compound 10	-6.993±0.165					✓
7DNC	SG85	-6.854±0.261					
	Compound 8v	-7.774±0.276					
	Compound 8w	-7.660±0.314					
	Compound 8x	-7.778±0.220					
	DC08090	-6.375±0.050					✓
	NK-1.8k	-7.370±0.281					✓
	NK-1.9k	-7.402±0.309					
	Compound 9	-7.284±0.314					✓
	FIOMC	-7.918±0.234					
	FOPMC	-7.647±0.191					
	GC376	-6.598±0.298					
