# Supplementary material for: Molecular docking study of various Enterovirus—A71 3C protease proteins and their potential inhibitors
Source: Front Microbiol. 2022 Sep 29;13:987801. doi: 10.3389/fmicb.2022.987801 (PMC9563145; doi:10.3389/fmicb.2022.987801)
Supplement: Supplementary file 5 [file Data_Sheet_5.pdf]

Supplementary S05. Molecular dynamic simulation of ligand-protein system using Gromacs tool with detail setting parameters.

Topology generation	Force field: AMBER99SB-ILDN protein, nucleic AMBER94<br>H atoms in the PDB file: Ignore<br>Water model: TIP3<br>NaCl concentration: 0.1M
Energy minimization	integrator = steep<br>emtol = 1000.0<br>emstep = 0.01<br>nsteps = 50000<br>nstlist = 1<br>cutoff-scheme = Verlet<br>ns_type = grid<br>rlist = 1.2<br>coulombtype = PME<br>rcoulomb = 1.2<br>vdw-modifier = force-switch<br>rvdw-switch = 1.0<br>rvdw = 1.2<br>pbc = xyz<br>DispCorr = no
Temperature equilibration	title = Protein-ligand complex NVT equilibration<br>define = -DPOSRES<br>integrator = md<br>nsteps = 50000<br>dt = 0.002<br>nstenergy = 500<br>nstlog = 500<br>nstxout-compressed = 500<br>continuation = no<br>constraint_algorithm = lincs<br>constraints = h-bonds<br>lincs_iter = 1<br>lincs_order = 4<br>cutoff-scheme = Verlet<br>ns_type = grid<br>nstlist = 20<br>rlist = 1.2<br>vdwtype = cutoff<br>vdw-modifier = force-switch<br>rvdw-switch = 1.0<br>rvdw = 1.2<br>coulombtype = PME<br>rcoulomb = 1.2<br>pme_order = 4<br>fourierspacing = 0.16<br>tcoupl = V-rescale<br>tc-grps = Protein_LIG Water_and_ions<br>tau_t = 0.1 0.1<br>ref_t = 300 300<br>pcoupl = no

	pbc = xyz<br>DispCorr = no<br>gen_vel = yes<br>gen_temp = 300<br>gen_seed = -1
Pressure equilibration	title = Protein-ligand complex NPT equilibration<br>define = -DPOSRES<br>integrator = md<br>nsteps = 50000<br>dt = 0.002<br>nstenergy = 500<br>nstlog = 500<br>nstxout-compressed = 500<br>continuation = no<br>constraint_algorithm = lincs<br>constraints = h-bonds<br>lincs_iter = 1<br>lincs_order = 4<br>cutoff-scheme = Verlet<br>ns_type = grid<br>nstlist = 20<br>rlist = 1.2<br>vdwtype = cutoff<br>vdw-modifier = force-switch<br>rvdw-switch = 1.0<br>rvdw = 1.2<br>coulombtype = PME<br>rcoulomb = 1.2<br>pme_order = 4<br>fourierspacing = 0.16<br>tcoupl = V-rescale<br>tc-grps = Protein_LIG Water_and_ions<br>tau_t = 0.1 0.1<br>ref_t = 300 300<br>pcoupl = Berendsen<br>pcoupltype = isotropic<br>tau_p = 2.0<br>ref_p = 1.0<br>compressibility = 4.5e-5<br>refcoord_scaling = com<br>pbc = xyz<br>DispCorr = no<br>gen_vel = no
Molecular dynamics	title = Protein-ligand complex MD simulation<br>integrator = md<br>nsteps = 50000000<br>dt = 0.002<br>nstenergy = 50000<br>nstlog = 50000<br>nstxout-compressed = 50000<br>continuation = yes<br>constraint_algorithm = lincs<br>constraints = h-bonds

	lincs_iter = 1<br>lincs_order = 4<br>cutoff-scheme = Verlet<br>ns_type = grid<br>nstlist = 20<br>rlist = 1.2<br>vdwtype = cutoff<br>vdw-modifier = force-switch<br>rvdw-switch = 1.0<br>rvdw = 1.2<br>coulombtype = PME<br>rcoulomb = 1.2<br>pme_order = 4<br>fourierspacing = 0.16<br>tcoupl = V-rescale<br>tc-grps = Protein_LIG Water_and_ions<br>tau_t = 0.1 0.1<br>ref_t = 300 300<br>pcoupl = Parrinello-Rahman<br>pcoupltype = isotropic<br>tau_p = 2.0<br>ref_p = 1.0<br>compressibility = 4.5e-5<br>pbc = xyz<br>DispCorr = no<br>gen_vel = no
